# A study on the siting of emergency medical facilities under uncertain demand—A case study of Wuhan country parks

**DOI:** 10.3389/fpubh.2025.1504694

**Published:** 2025-03-21

**Authors:** Shuai Li, Wenli Chen, Zheng Wu, Jiefang Tang, Jiangtao Jiu, Pengfei Wang

**Affiliations:** ^1^College of Landscape Architecture and Art, Henan Agricultural University, Zhengzhou, China; ^2^College of Architecture and Urban Planning, Tongji University, Shanghai, China

**Keywords:** urban green space, public health, emergency medical facility, robust optimization, country parks

## Abstract

**Introduction:**

At the beginning of 2020, the novel coronavirus broke out as a sudden public health emergency worldwide, with the number of confirmed patients constantly rising, which brought huge pressure to the medical system. Many countries and regions have noticed the positive role of emergency medical facilities in combating the COVID-19 pandemic. Therefore, the analysis of the location and construction of emergency medical facilities for public health emergencies has practical significance.

**Objective:**

This paper mainly discusses the use of urban suburban parks as the construction sites for emergency medical facilities and builds a maximum service quality level model for emergency medical facilities in response to public health emergencies.

**Method:**

Considering the suddenness and unpredictability of public health emergencies, this study introduces polyhedral uncertainty sets to describe the uncertainty of the number of confirmed patients and transforms the model into an easily solvable mixed-integer programming model through the Bertsimas and Sim robust optimization method. The GAMS software is used for programming and the CPLEX solver is called to solve the model. Taking 13 urban suburban parks in Wuhan as an example, the optimal location plan and patient allocation of emergency medical facilities are determined, verifying the feasibility and effectiveness of the model.

**Discussion:**

The results show that the model effectively promotes the determination of location plans and patient transfer routes. It is expected that in the event of a sudden public health emergency in a city, it can provide reference and basis for decision-makers to deal with public health emergencies.

## 1 Introduction

In December 2019, an acute respiratory infectious disease caused by the SARS-CoV-2 virus first broke out in Wuhan, China, and rapidly spread worldwide. Subsequently, the epidemic spread to over 200 countries and regions around the globe, posing a serious threat to social and economic development. It was defined by the World Health Organization (WHO) as a serious global public health emergency (1). After the diagnosis of COVID-19, most infected people have mild symptoms, such as dry cough, sore throat and fever. However, some patients have fatal outbreaks such as organ failure and severe pneumonia (2, 3). Research has shown that the SARS-CoV-2 virus is mainly transmitted through respiratory droplets, aerosols, and direct or indirect contact, with an average incubation period of about 5 days (4, 5). These Pathogen transmission and latent characteristics led to the rapid spread of the COVID-19 in a short period of time, posing a great threat and challenge to the global public health security system (6, 7).

The outbreak of the COVID-19 has led to insufficient supply of medical facilities, shortage of medical resources, difficulty in receiving patients and other situations in many regions. The key to deal with the epidemic of large-scale infectious diseases is to improve the admission and cure rate of patients. Building emergency medical facilities can play a crucial role in responding to sudden public health emergencies when existing designated treatment hospitals cannot meet the treatment needs (8, 9). For example, referring to the Xiaotangshan Hospital model established by Beijing in 2003 to fight against the SARS epidemic, Wuhan has successively built Huoshenshan Hospital and Leishenshan Hospital to treat and isolate patients with COVID-19 (10, 11). In addition to Wuhan, large-scale transmission of the COVID-19 has occurred in many regions of the world (12). Several countries have begun to build field hospitals and mobile cabin hospitals to combat the COVID-19. For example, in the United States, Spain, Britain, Italy and other countries, the existing public buildings such as conference halls, exhibition centers, stadiums and concert halls have been transformed into emergency medical facilities (13–16). The location selection of emergency medical facilities is crucial. These countries and regions have decided to transform existing public buildings into emergency medical facilities because they meet the renovation standards and have infrastructure such as water and power supply (17). Some countries have also built emergency medical facilities in outdoor public spaces. For example, the emergency hospital in New York City is built within Central Park (18). In Malaysia, country agricultural sightseeing parks were converted into emergency medical facilities (19). However, SARS-CoV-2 virus spread faster indoors than outdoors, and indoor virus particle concentration is often higher than outdoor (20). It can be seen that it is very important to ensure the safety, rationality and scientific of emergency medical facility site selection when providing treatment for patients with COVID-19 infection.

The World Health Organization mentioned in the Urban green space and Health Report that Urban green space can stimulate social cohesion, support sports activities, reduce exposure to air pollutants, noise, etc., promote the physical and mental health of urban residents, and reduce incidence rate and mortality by providing psychological relaxation and relieving pressure (21).

Since the COVID-19 epidemic spread around the world, many researchers have reconsidered the relationship between Urban Green Space (UGS) and public health. Liu Lu analyzed the spread of COVID-19 from an urban perspective, and mentioned that urban green open space can play a positive role in building temporary hospitals and providing recreational and leisure functions (22). Starting from the concept of “combining peacetime and war,” Lu Ming put forward a mixed-nature land use model that divides urban reserve land into two target types: “wartime” medical and health land and “peacetime” park and green space (23). Jordi Honey-Roses et al. suggest that existing types and functions of green space should be revisited in the context of the COVID-19 pandemic (24). Tang Jiefang et al. proposed that country parks were highly compatible with the location of emergency medical facilities, and demonstrated that the former was a more ideal type of green space to avoid epidemics (25). Green parks are indispensable in urban disaster prevention planning, and country parks play an important role in coping with public health emergencies in disaster mitigation because of their special advantages (26).

As an important means to control the spread of the epidemic, the scientific location and timeliness of the construction of emergency medical facilities have also played an important role (27). Zhou et al. analyzed the spatial layout of EMS facilities in Beijing through the network location allocation model, and found that EMS demand is affected by changes in time and region, and traffic data is crucial to response time (28). Deng et al. optimized the EMS facility layout in Chengdu by using genetic algorithm and location set coverage model to improve coverage and system efficiency (29). Jin et al. studied the layout of emergency medical facilities in Wuhan and found that the “one center and two circles” structure can effectively improve the accessibility and service coverage of residents (30). Chen et al. further noted the importance of Shelter Hospital in emergency response, supporting the urban resilience emergency response framework (31). Liu et al. proposed a location model of EMS facilities in Guangzhou based on MATLAB genetic algorithm to optimize resource allocation (32). Taking the site selection of emergency temporary hospitals in Istanbul, Turkey during the COVID-19 pandemic as an example, Chang compared three site selection methods: flexible emergency location selection method, spherical weighted arithmetic average method and spherical aggregation operator method (33). However, most of the optimization objectives of fair and balanced allocation of emergency service resources are based on deterministic demand or supply, and the research method of deterministic model cannot be applied to the decision-making situation of uncertain demand or supply of emergency epidemic (34).

The location of emergency medical facilities is faced with many uncertain factors, such as the number of patients, types of diseases and scale of emergencies, and the changes of these factors are difficult to predict accurately. Therefore, how to choose the location of facility construction under uncertain environment has become a key problem to be solved urgently. With the rapid development of robust optimization methods, the application of robust optimization theory to solve the uncertainty of facility location has become a hot research topic. Liu et al. propose a distributed robust model that optimizes the facility location and number of ambulances in an EMS system to cope with uncertainty in demand (35). Razavi et al. investigated transfusion networks in post-disaster field hospitals and proposed a multi-objective robust optimization model designed to optimize blood type and blood distribution routes under uncertain conditions (36). Sun et al. proposed a scenario-based robust dual-objective optimization model that integrates medical facility location, casualty transportation, and material distribution, and validated its effectiveness in the Wenchuan earthquake (37). Wang et al. propose a Location Assignment problem (LAP) model to help emergency medical facilities (EMFs) respond to major public health emergencies. Taking into account the impact of the number of COVID-19 infections on EMF demand, they also used a gray prediction model to predict cumulative cases during the pandemic and calculate EMF demand (38). Yuan et al. used distributed robust chance constrained programming to study the location and size problems of emergency medical service systems and described them in an ambiguity set constructed based on Wasserstein metrics (39). Xu et al. combined entropy weight method and robust optimization method to optimize EMS location of hierarchical diagnosis and treatment system (40). These studies play an obvious role in rational allocation of medical resources and improving utilization efficiency of medical resources. Country parks are highly compatible with the location of emergency medical facilities, but the existing robust optimization studies rarely discuss the uncertainty of using urban green space as emergency shelter from the perspective of urban green space.

Therefore, unlike previous studies, this research takes the disaster prevention and emergency shelter function of urban public green spaces as the starting point, and selects the suburban parks located at the edge of the city as the construction sites for emergency medical facilities. Combined with the robust optimization theory, it constructs an emergency medical facility location optimization model based on uncertain demand, aiming at the pre-epidemic prevention and preparation and post-epidemic patient treatment needs of the COVID-19 epidemic. This model particularly considers the differentiated demands of different patient types for medical facilities, the number of patients, the transfer time, the distance and other research variables, which to a certain extent affect the rationality of resource allocation and the treatment efficiency of patients. Taking the COVID-19 epidemic in Wuhan as an example, the reliability and robustness of the proposed model are verified, providing new ideas for the construction of urban emergency medical facilities and the allocation of emergency resources.

## 2 Research object and research method

### 2.1 Overview of the research area

**Figure 1:**
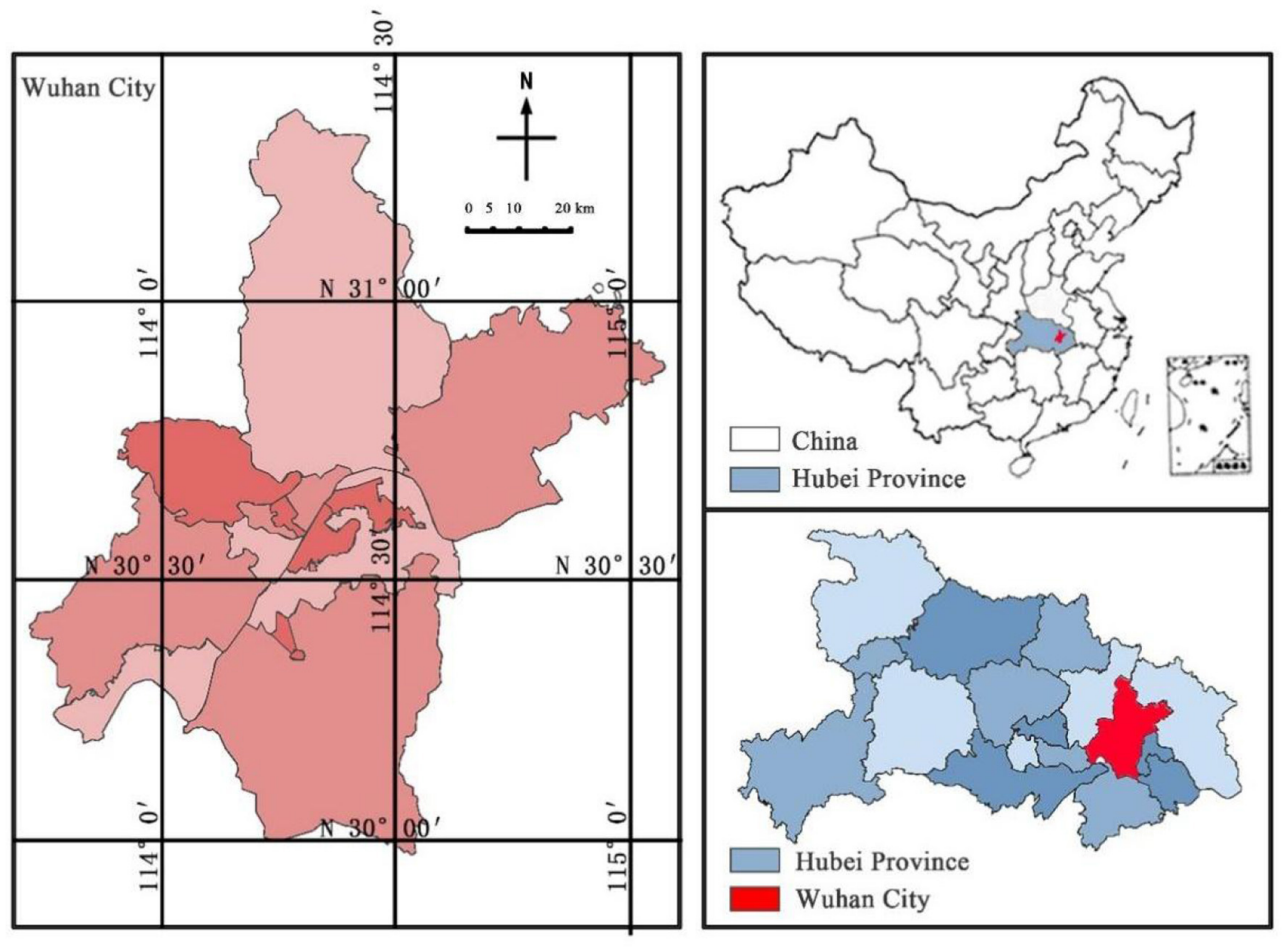
Location of Wuhan.

Wuhan is located in the south-central region of China, in the *Jianghan* Plain at the middle reaches of the Yangtze River. It serves as the capital city of Hubei Province and is one of six central provinces (Figure 1). Covering a total area of 8569.15 km^2^, Wuhan has jurisdiction over 13 districts and boasts a permanent population exceeding 13.739 million people. As China's largest transportation hub for water, land, and air travel, it plays an integral role in connecting various regions throughout the country. The huge population size and high frequency mobility are the key to the rapid spread of the epidemic (41).

At the beginning of 2020, COVID-19 broke out in Wuhan, and within <1 month (from 20 January 2020 to 17 February 2020), the number of confirmed infected persons surged to more than 40,000. Faced with the huge emergency demand for prevention and control, medical resources in Wuhan are in short supply. In order to concentrate the advantageous medical resources to rescue the critically ill patients, the Wuhan Municipal Government quickly launched two emergency medical facilities, *Huoshenshan* Hospital and *Leishensha*n Hospital, and accessed the first batch of patients respectively on February 4 and 8. On 15 April 2020, as the epidemic situation in Wuhan stabilized, the two hospitals successively closed their isolation wards, which served as a symbol of Wuhan's significant phased achievements in combating COVID-19 (42).

### 2.2 Data source

The data sources include the Notice of Wuhan Natural Resources and Planning Bureau on Releasing the Wuhan City Map in 2021 issued by Wuhan Natural Resources and Planning Bureau, the country park list listed in the Wuhan City Post-Epidemic Revitalization Plan (Three-year Action Plan) issued by Wuhan City Planning and Research Institute in 2021, and the Wuhan Statistical Year issued by Wuhan City Statistics Bureau in 2023 And the Statistics Table of Major urban Parks in Wuhan issued by Wuhan Municipal Bureau of Garden and Forestry. The country park of Wuhan was taken as the research object, and the administrative boundary of Wuhan was taken as the research boundary, involving an area of 8569.15 km^2^. According to the list of country parks in the “Wuhan Post-Epidemic Revitalization Plan (Three-year Action Plan),” considering that Wuhan has a history of more than 3,500 years of urban construction, the ground and underground materials are abundant, which belong to non-renewable human resources, and many wetland parks are connected to the Yangtze River water system, improper control is easy to cause water pollution, and there is a risk of waterborne transmission (43). Therefore, country parks with cultural and wetland resources are not suitable as types of emergency medical facilities for construction. After screening, 13 country parks in Wuhan were identified as the research subjects for this study. With reference to Ovi Interactive Map, Google Maps Satellite imagery and Baidu Maps, the location, area and other information of 13 country parks were corrected by using the registration domain digitization function of ArcGIS10.5 (Table 1).

**Table 1:** List of country parks.

**Park number**	**Name of country park**	**Area of country park (hm^2^)**
1	Baiquan Country Park	2041.23
2	Mulan Hill Country Park	5862.53
3	Cangbu Country Park	1776.46
4	Zhangdu Lake Country Park	6799.5
5	Yan Donghu Country Park	1637.61
6	Longquan Mountain Country Park	1767.88
7	Bafenshan Country Park	1194.67
8	Longling Mountain Country Park	298.79
9	Bijia Mountain Country Park	150.6
10	Agricultural New Year Country Park	171.74
11	Huashan Lake Country Park	460.64
12	Changling Mountain Country Park	126.06
13	Jinlong Country Park	1079.18

### 2.3 Research method

After the outbreak of COVID-19, the number of diagnosed patients increased exponentially (3). Due to the limited number and capacity of designated treatment hospitals, their ability to treat patients is not as fast as the increase in the number of diagnosed patients. In order to improve emergency work efficiency, diagnosed patients are divided into two categories based on their urgency level: One type of patients are critically ill patients who need to enter the ICU in a timely manner and receive ECMO (Extracorporeal Membrane Oxygen) treatment (44, 45); The other type of patients are mildly ill patients, mainly with symptoms of upper respiratory tract infection, requiring isolation treatment, disease monitoring, etc. To ensure prompt treatment for all patients, emergency medical facilities are categorized into two types: the first type is designed to treat critically ill or severely injured patients, while the second type provides care for those with mild symptoms. Considering the varying degrees of spatial fragmentation and different infrastructure conditions in various country parks, it is assumed that only one emergency medical facility will be constructed in the same country park.

The service quality of emergency medical facilities is an important factor in measuring rescue effectiveness. The service quality coverage function is introduced to represent the mathematical relationship between service coverage level and distance (Equation 1) (46). Describe the service quality (q) of emergency medical facilities through the distance function between the coverage level of emergency medical facilities in designated treatment hospitals and the distance between designated treatment hospitals and country parks.


(1) $$q=\{\begin{array}{c} 1,\;d_{ij}\leq d_{\min}\\ e^{-a{\frac{\left(d_{ij}-d_{\min}\right)}{\left(d_{\max}-d_{\min}\right)}}^{b}},\;d_{\min}\leq d_{ij}\leq d_{\max}\\ 0,\;d_{ij}\geq d_{\max} \end{array}$$

In Equation 1, *d*_*ij*_ represents the distance from designated treatment hospital *i* to country park *j*, *d*_min_ represents the minimum critical distance that a designated treatment hospital is covered by emergency medical facilities, *d*_max_ represents the maximum critical distance that a designated treatment hospital is covered by emergency medical facilities; *a* and *b* represent Exponential decay constants, which are used to reflect the shape and decay of the function.

Therefore, the problem addressed in this article pertains to the selection of construction sites for emergency medical facilities within country parks under conditions of uncertain patient numbers and with consideration given to service coverage across multiple patient types, all with the aim of maximizing overall weighted service quality levels (47). The relevant parameters are explained as follows:

I: The set of designated treatment hospitals, denoted by *i*, *i* ∈ *I*;
J: The set of country parks, denoted by *j*, *j* ∈ *J*;
K: The set of diagnosed patient types, denoted by *k*, *k* ∈ *K*; *k* = 1 represents critically ill patients, and *k* = 1 represents mildly ill patients;
L: The set of emergency medical facility types, denoted by *l*, *l* ∈ *L*; *l* = 1 represents rescue type, *l* = 1 represents monitoring type;
*c*_*l*_: The maximum admission capacity of emergency medical facilities of type *l*;
*p*_*ik*_: The number of patients of type *k* at designated treatment hospital *i*;
*d*_*ij*_: The distance from designated treatment hospitals *i* to emergency medical facility *j*;
*d*_min_: The minimum critical distance that a designated treatment hospital is covered by emergency medical facilities;
*d*_max_: The maximum critical distance that a designated treatment hospital is covered by emergency medical facilities;
*n*_*l*_: The number of emergency medical facilities of type *l*;
*w*_*l*_: The weight of emergency medical facilities of type *l*;
*f*_*ikj*_: The level of coverage of the emergency medical facilities at country park *j* for type *k* patients at designated treatment hospital *i*.

**Decision variables:**

*x*_*jl*_: x is a binary variable. If emergency medical facilities of type *l* are established at location *j* in a country park, then *x*_*jl*_=1; Otherwise, *x*_*jl*_=0;
*y*_*ikjl*_: y is a positive variable, representing the proportion of type *k* patients in designated treatment hospitals *i* covered by the emergency medical facilities established at *j* in the country park.

#### 2.3.1 Normal model

When the number of patients of type *k* at designated treatment hospital *i* is known, the nominal model (DM) is as follows:


(2) $$\begin{array}{l} \left(\text{DM}\right)Z^{D}=\max\underset{i\in I}{\sum }\underset{k\in K}{\sum }\underset{j\in J}{\sum }\underset{l\in L}{\sum }w_{l}p_{ik}f_{ikj}y_{ikjl} \end{array}$$

(3) $$\begin{array}{l} s.t.\;\underset{i\in I}{\sum }\underset{k\in K}{\sum }p_{ik}y_{ikjl}\leq c_{l},\forall j\in J,l\in L \end{array}$$

(4) $$\begin{array}{l} y_{ikjl}\leq x_{jl},\forall i\in I,j\in J,k\in K,l\in L \end{array}$$

(5) $$\begin{array}{l} \underset{j\in J}{\sum }x_{jl}\leq n_{l},\forall l\in L \end{array}$$

(6) $$\begin{array}{l} \underset{l\in L}{\sum }x_{jl}\leq 1,\forall j\in J \end{array}$$

(7) $$\begin{array}{l} \underset{j\in J}{\sum }\underset{l\in L}{\sum }y_{ikjl}\leq 1,\forall i\in I,k\in K \end{array}$$

(8) $$\begin{array}{l} y_{ikjl}=0,\forall i\in I,j\in J,k=2,l=1\;or\;k=1,l=2\; \end{array}$$

(9) $$f_{ijl}=\{\begin{array}{c} 1,\;d_{ij}\leq d_{\min}\\ e^{-a{\frac{\left(d_{ij}-d_{\min}\right)}{\left(d_{\max}-d_{\min}\right)}}^{b}},\;d_{\min}\leq d_{ij}\leq d_{\max}\\ 0,\;d_{ij}\geq d_{\max} \end{array}\;,\forall i\in I,j\in J,l\in L$$

(10) $$\begin{array}{l} x_{jl}\in \left\{0,1\right\},y_{ikjl}\geq 0,\forall i\in I,k\in K,j\in J,l\in L\; \end{array}$$

ss In the nominal model, the objective function Equation 2 represents the maximum total weighted service quality level of emergency medical facilities. Constraint Equation 3 represents the constraint on the ability of emergency medical facilities to accommodate patients. Constraint Equation 4 indicates that only emergency medical facilities can be established to provide services. Constraint Equation 5 indicates that the number of emergency medical facilities is limited and cannot exceed the maximum construction quantity. Constraint Equation 6 indicates that at most one emergency medical facility can be established in the same country park. Constraint Equation 7 indicates that the sum of the proportion of allocated patients does not exceed 1. Constraint Equation 8 indicates that mildly ill patients cannot be transported to rescue type emergency medical facilities, critically ill patients cannot be transported to monitoring type emergency medical facilities. Constraint Equation 9 is the expression for the service quality level of emergency medical facilities. Constraint Equation 10 represents the constraint of the decision variable.

#### 2.3.2 Robust optimization model

When the COVID-19 broke out, the number of infected patients fluctuated greatly due to the limited capacity of designated treatment hospitals, and there was obvious uncertainty in the number of patients who were isolated for observation and clinical treatment. Therefore, based on the nominal model mentioned above, the Polyhedral uncertainty set is introduced. The number of patients with transport type *k* required for designated treatment hospital *i* is ${\tilde{p}}_{ik}$, and ${\tilde{p}}_{ik}\in \left[p_{ik}-{\hat{p}}_{ik}\varphi_{ik},p_{ik}+{\hat{p}}_{ik}\varphi_{ik}\right]$. *p*_*ik*_ is the number of patients with transport type *k* required for designated treatment hospitals *i* in the nominal model, ${\hat{p}}_{ik}$ is the perturbation momentum, uncertain set is $\text{Ψ}=\left\{\varphi :\underset{i\in I}{\sum }\varphi_{ik}\leq {\text{Γ}}_{k},\forall k\in K,\;0\leq \varphi_{ik}\leq 1\right\}$, where Γ_*k*_ represents the uncertainty level of the uncertain set, which is used to objectively measure the conservatism of constraint conditions and reflect the risk preference of decision-makers. The smaller the value of Γ_*k*_, the higher the decision-maker's preference for risk pursuit. The Robust Optimization Location Model (RM) is as follows:


(11) $$\begin{array}{l} \left(\text{RM}\right)Z^{R}=\max\left\{\underset{\varphi \in \text{Ψ}}{\min}\sum_{i\in I}\sum_{k\in K}\sum_{j\in J}\sum_{l\in L}w_{k}\left(p_{ik}+{\hat{p}}_{ik}\varphi_{ik}\right)f_{ijl}y_{ikjl}\right\}\\ \;s.t. \end{array}$$

Equations 4–10


(12) $$\begin{array}{l} \;\underset{\varphi \in \text{Ψ}}{\max}\sum_{i\in I}\sum_{k\in K}\left(p_{ik}+{\hat{p}}_{ik}\varphi_{ik}\right)y_{ikjl}\\ =\sum_{i\in I}\sum_{k\in K}p_{ik}y_{ikjl}+\underset{\varphi \in \text{Ψ}}{\max}\sum_{i\in I}\sum_{k\in K}{\hat{p}}_{ik}\varphi_{ik}y_{ikjl}\leq c_{l},\\ \;\forall j\in J,l\in L \end{array}$$

When Γ_*k*_ = 0, the robust model is equivalent to the nominal model. There is an inner layer minimization problem in the objective function (Equation 10), which will be transformed into a more easily solvable robust equivalent model.


(13) $$\begin{array}{l} max\left\{\underset{\varphi \in \text{Ψ}}{\min}\sum_{i\in I}\sum_{k\in K}\sum_{j\in J}\sum_{l\in L}w_{k}\left(p_{ik}+{\hat{p}}_{ik}\varphi_{ik}\right)f_{ijl}y_{ikjl}\right\}\\ =\max\{\sum_{i\in I}\sum_{k\in K}\sum_{j\in J}\sum_{l\in L}w_{k}p_{ik}f_{ijl}y_{ikjl}\\ \;+\underset{\varphi \in \text{Ψ}}{\min}\sum_{i\in I}\sum_{k\in K}\sum_{j\in J}\sum_{l\in L}w_{k}{\hat{p}}_{ik}\varphi_{ik}f_{ijl}y_{ikjl}\}\\ s.t. \end{array}$$

Equations 4–10, 12

Consider the Linear programming problem of inner minimization:


(14) $$\begin{array}{r} \underset{\varphi \in \text{Ψ}}{\min}\sum_{i\in I}\sum_{k\in K}\sum_{j\in J}\sum_{l\in L}w_{k}{\hat{p}}_{ik}\varphi_{ik}f_{ijl}y_{ikjl}\\ s.t.\sum_{i}\sum_{k}\varphi_{ik}\leq {\text{Γ}}_{k}\\ 0\leq \varphi_{ik}\leq 1,\forall i\in I,k\leq K \end{array}$$

According to the principle of duality, problem Equation 14 can be equivalent to problem Equation 15, where α_*ik*_, β_*k*_ are dual variables. Question Equation 12 is the same.


(15) $$\begin{array}{l} \min\underset{i\in I}{\sum }\underset{k\in K}{\sum }\alpha_{ik}+\underset{k\in K}{\sum }\beta_{k}{\text{Γ}}_{k} \end{array}$$

(16) $$\begin{array}{l} s.t.\;\alpha_{ik}+\beta_{k}\leq \underset{j\in J}{\sum }\underset{l\in L}{\sum }w_{k}{\hat{p}}_{ik}{f_{ijl}y}_{ikjl},\forall i\in I,k\in K \end{array}$$

(17) $$\begin{array}{l} \alpha_{ik},\beta_{k}\geq 0,\forall i\in I,k\in K\; \end{array}$$

In summary, problems Equation 12 and Equation 14 are substituted into problem Equation 11, and based on the robust architecture proposed by Bertsimas and Sim (48). The Robust Optimization model (RM) is transformed into an Equivalent Mixed Integer Programming Model (EM).


(18) $$\begin{array}{l} \left(EM\right)Z^{E}=\max\underset{i\in I}{\sum }\underset{k\in K}{\sum }\underset{j\in J}{\sum }\underset{l\in L}{\sum }w_{k}p_{ik}{f_{ijl}y}_{ikjl}+\xi \\ \;s.t. \end{array}$$

Equations 4–10, 16, 17,


(19) $$\begin{array}{l} \xi \leq \underset{i\in I}{\sum }\underset{k\in K}{\sum }\alpha_{ik}+\underset{k\in K}{\sum }\beta_{k}{\text{Γ}}_{k} \end{array}$$

(20) $$\sum_{i\in I}\sum_{k\in K}p_{ik}y_{ikjl}+\sum_{i\in I}\sum_{k\in K}\alpha_{ik}\prime +\sum_{k\in K}\beta_{k}\prime \Gamma_{k}\leq c_{l},\forall j\in J,l\in L$$

(21) $${\alpha_{ik}}^{\prime }+{\beta_{k}}^{\prime }\geq \sum_{j\in J}\sum_{l\in L}{\hat{p}}_{ik}y_{ikjl},\forall i\in I,k\in K$$

(22) $${\alpha_{ik}}^{\prime },{\beta_{k}}^{\prime },\geq 0,\forall i\in I,k\in K$$

Among them, ξ is the auxiliary variable, α_*ik*_, β_*k*_ are the dual variables of problem Equation 14, $\alpha_{ik}^{\prime },\beta_{k}^{\prime }$ are the dual variables of problem Equation 12.

Therefore, on the basis of the nominal model, considering the uncertainty of the number of diagnosed patients transported *p*_*ik*_, a polyhedral uncertainty set is introduced to characterize the uncertainty. With the help of robust optimization theory, the established robust location optimization model is transformed into a mixed linear integer programming model that is easy to solve and handle, which can be solved using existing mathematical software. This article uses GAMS 28.2 to program the model and calls the Branch-Cut Algorithm in the CPLEX 12.9 solver to solve it.

### 2.4 Example analysis

**Figure 2:**
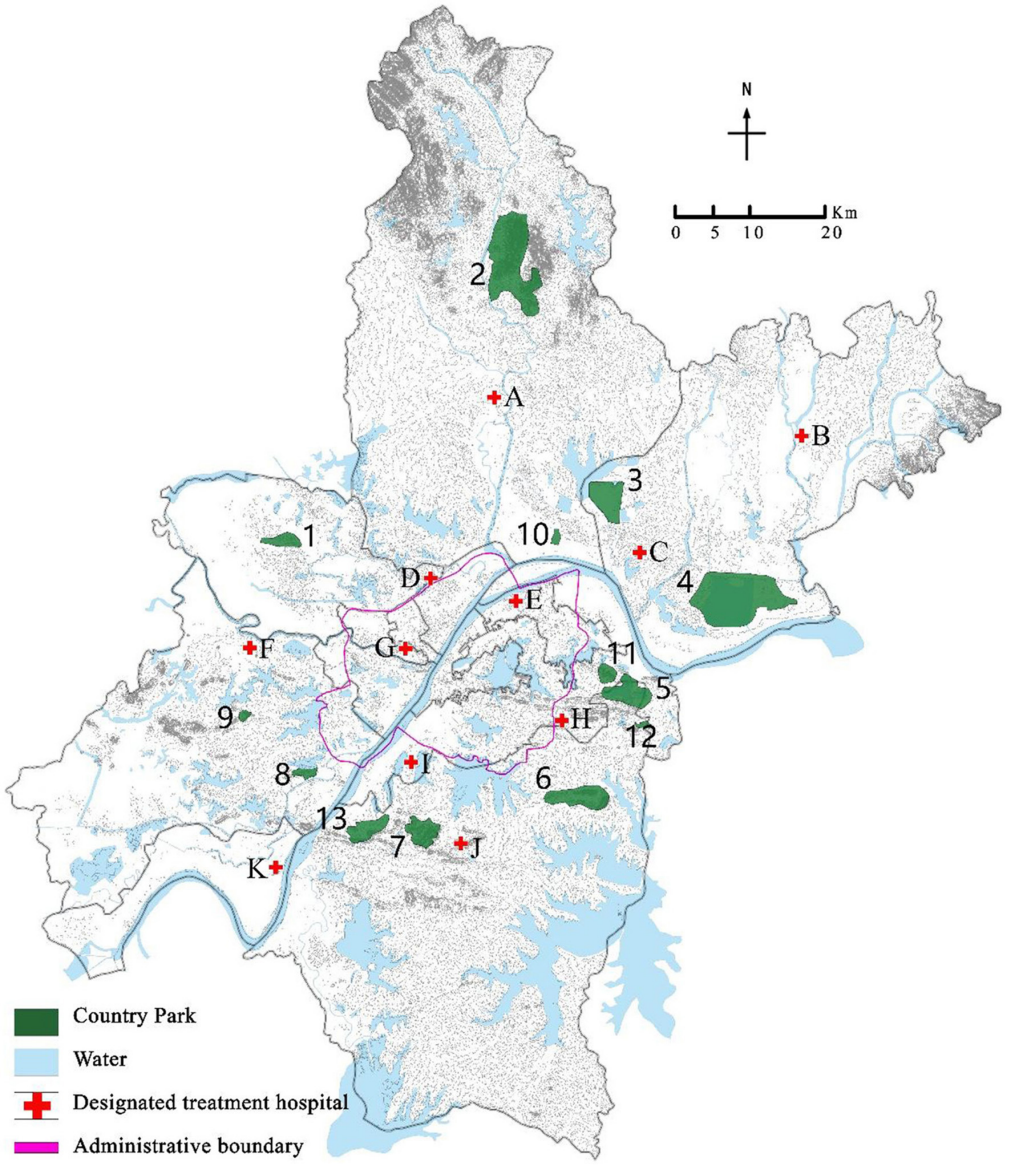
Geographical location of designated treatment hospitals and country parks.

In this paper, 11 designated treatment hospitals of COVID-19 in each district of Wuhan City, such as Huangpi District People's Hospital, Wuhan Lung Hospital, Wuhan Xincheng Hospital, are selected as the designated treatment hospital set *I* (Table 2), and 13 country parks, such as Baiquan Country Park, Mulan Mountain Country Park, Cangbu Country Park, are selected as the alternative address set *J* for emergency medical facilities. The geographical relationship and serial number of the designated treatment hospitals and country parks are shown in Figure 2. For the convenience of expression, serial numbers are used to represent each location in the following text.

**Table 2:** List of designated treatment hospitals.

**Number**	**Name of designated treatment hospital**
A	Huangpi District People's Hospital
B	Xinzhou District Traditional Chinese Medicine Hospital
C	Wuhan Xincheng Hospital
D	Wuhan Jinyintan Hospital
E	Wuhan Iron and Steel Second Hospital
F	Caidian District Hospital of Traditional Chinese Medicine
G	Wuhan Pulmonary Hospital
H	Tongji Hospital Guanggu Hospital Area
I	Hospital of Wuhan University of Science and Technology
J	Jiangxia District Hospital of Traditional Chinese Medicine
K	Hannan District Hospital of Traditional Chinese Medicine

Based on the severity of the COVID-19 epidemic, the capacity of designated treatment hospitals for treatment, and the proportion of severe and mildly patients, the number of patients *p*_*ik*_ (nominal number of diagnosed patients) that need to be transported in each designated treatment hospitals is estimated (49), as shown in Table 3. The distance from designated treatment hospitals to country parks is shown in Table 4. At the beginning of the outbreak, two emergency medical facilities, *Leishenshan* Hospital and *Huoshenshan* Hospital, were built in Wuhan, and the three mobile cabin hospitals to receive patients with mildly illnesses were located in Wuhan Convention and Exhibition Center, *Hongshan* Stadium and Wuhan Living Room respectively. Therefore, it is assumed that a total of 5 emergency medical facilities will be constructed in this issue, including 2 for monitoring type and 3 for monitoring type (50). Referring to the admission capacity of *Leishenshan* Hospital, *Huoshenshan* Hospital and many mobile cabin hospitals in Wuhan during the use period, the admission capacity *c*_*l*1_ of rescue type emergency medical facilities is 1,600 persons, and the admission capacity *c*_*l*2_ of monitoring type emergency medical facilities is 3,000 persons (51). The longest transport time of patients with COVID-19 is not more than 2 h (52), Assuming the average speed of transfer vehicles is *v* = 30 *km*/*h*. The maximum critical distance covered by the service scope of the designated treatment hospital is *d*_max_ = 60, minimum critical distance *d*_min_ = 20. The importance coefficients of rescue and monitoring emergency medical facilities are 1 and 0.5, respectively. Take 2 from *a* and *b* to represent the attenuation of the function value between 0 and 1. When considering the uncertainty level parameter Γ_*k*_, it is assumed that the amplitude of change corresponding to each constraint is the same, i.e., Γ_*k*_ = Γ,and Γ is taken as an integer (53).

**Table 3:** Nominally confirmed patients in designated treatment hospitals (number).

	**i1**	**i2**	**i3**	**i4**	**i5**	**i6**	**i7**	**i8**	**i9**	**i10**	**i11**
Critically ill patients (person)	220	134	100	720	120	136	122	166	30	203	46
Mildly ill patients (person)	880	535	400	2,880	480	544	488	662	120	824	184

**Table 4:** Distance from designated treatment hospitals to country parks (km).

	**j1**	**j2**	**j3**	**j4**	**j5**	**j6**	**j7**	**j8**	**j9**	**j10**	**j11**	**j12**	**j13**
i1	39	24	48	61	64	75	72	74	69	31	58	68	86
i2	87	70	42	29	65	79	105	114	102	47	59	69	110
i3	69	74	15	23	32	47	73	67	71	16	26	37	78
i4	28	60	47	54	46	53	49	40	44	23	39	52	49
i5	51	67	42	43	29	37	50	48	52	19	23	36	56
i6	24	91	73	79	66	65	51	23	15	52	60	61	43
i7	26	71	50	56	47	46	36	29	35	29	40	42	36
i8	61	86	62	44	21	16	33	51	55	45	14	15	39
i9	54	95	71	79	45	37	17	29	39	57	38	40	14
i10	76	104	78	70	44	26	7	41	52	56	38	38	16
i11	63	122	94	105	86	64	37	20	31	73	80	75	30

## 3 Results

**Figure 3:**
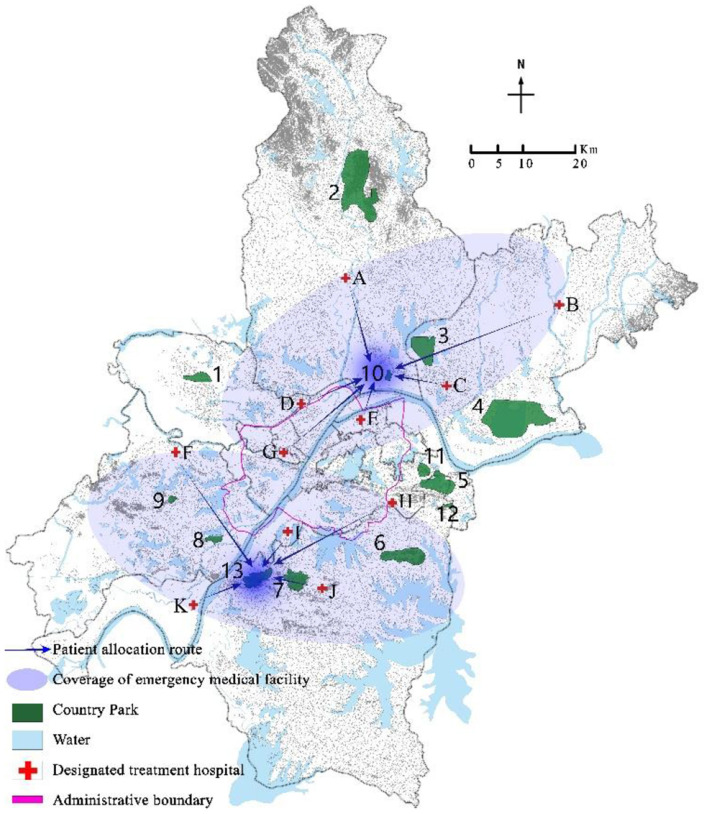
Coverage results of critically ill patients in the nominal model.

**Figure 4:**
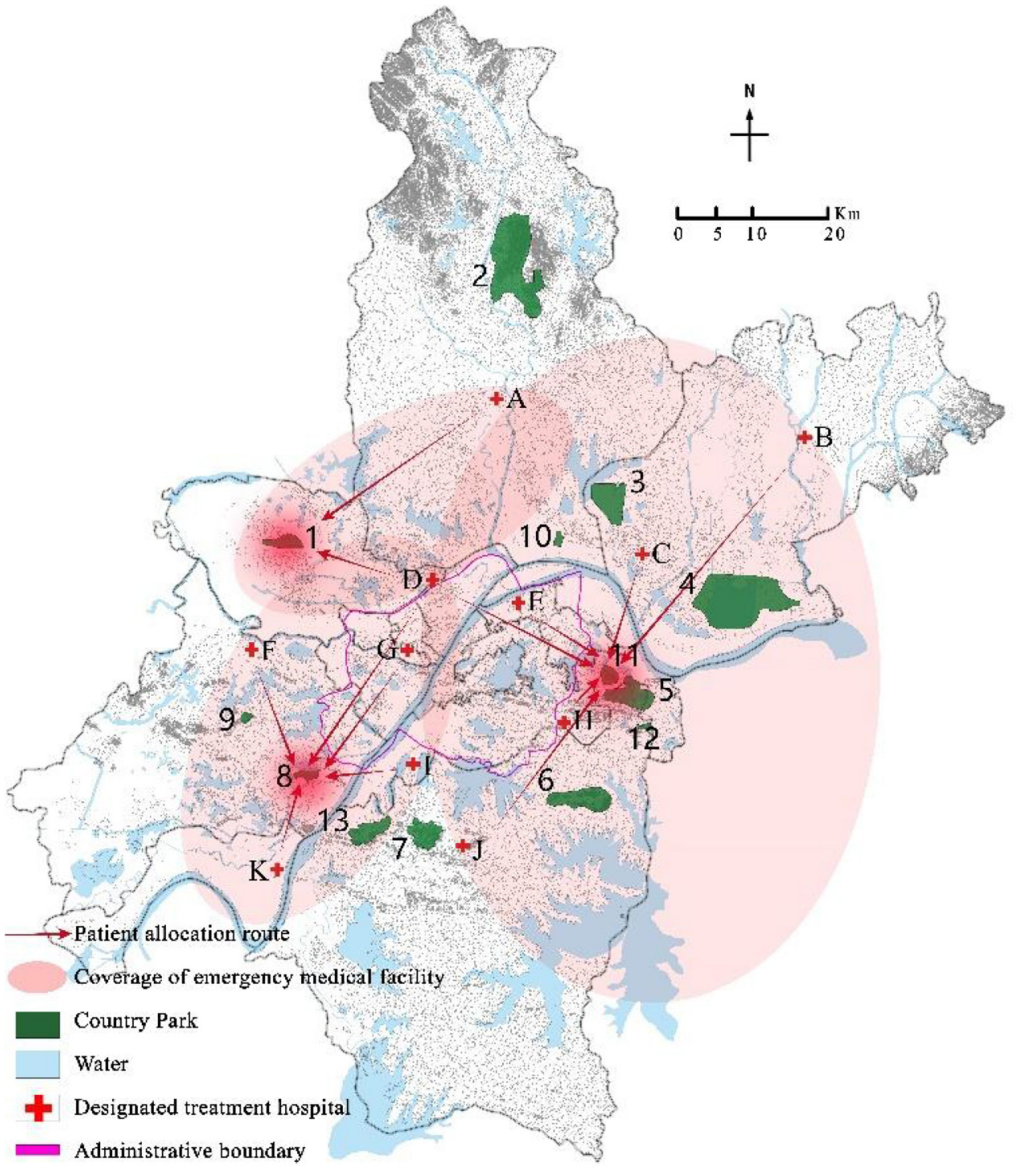
Coverage results of mildly ill patients in the nominal model.

**Figure 5:**
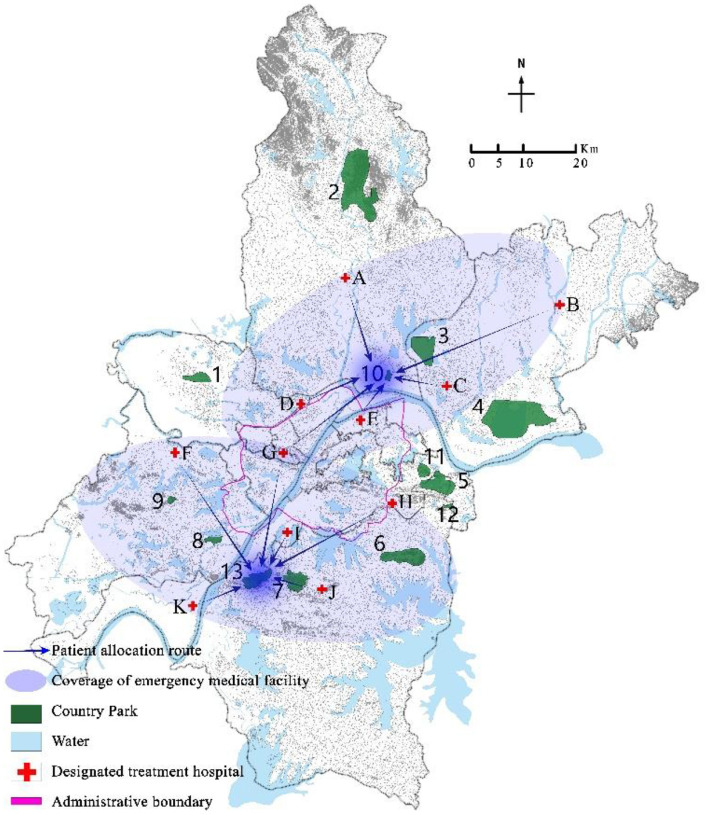
Coverage results of critically ill patients using a robust optimization model with a disturbance ratio of 5% and Γ = 5.

**Figure 6:**
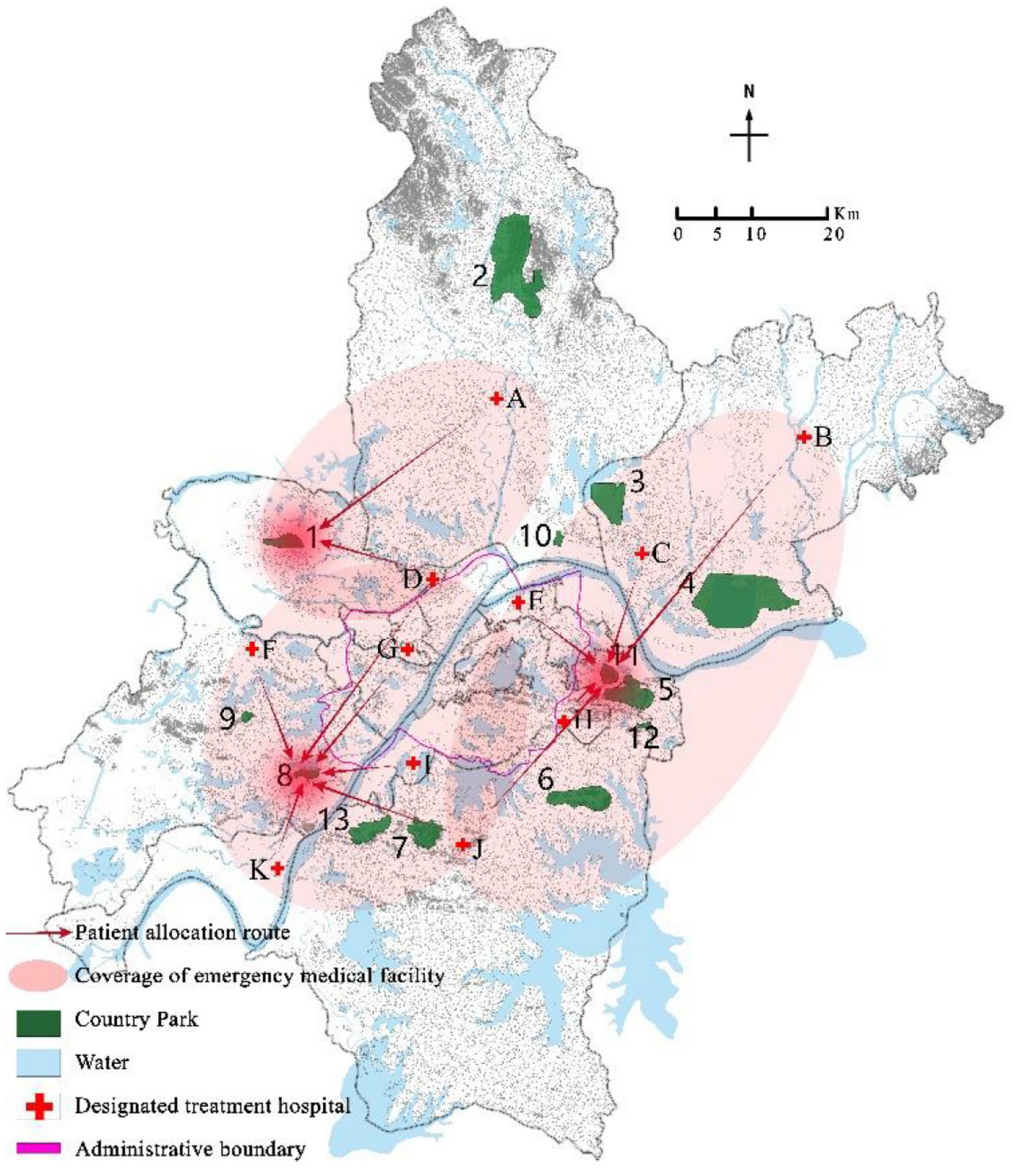
Coverage results of mildly ill patients using a robust optimization model with a disturbance ratio of 5% and Γ = 5.

Program the mixed integer programming model (EM) through GAMS 28.2 and call CPLEX 12.9 for solution. Select a disturbance ratio of 5%, and the uncertainty level parameter Γ = 5. The optimal location scheme and emergency medical facility coverage of robust optimization models under nominal model and polyhedral uncertainty set are shown in Figures 3–6 respectively. The circle represents the coverage range of emergency medical facilities construction, and the arrow represents patient coverage.

The model solution results show that both the nominal model and the robust optimization model choose to construct rescue type emergency medical facilities in country parks Nos. 10 and 13, and monitoring type emergency medical facilities in country parks Nos.1, 8, and 11. However, the allocation of emergency medical facilities to patients in the two models is different. For example, in the nominal model, 100% of critically ill patients in the designated treatment hospital No. 4 are covered by the service scope of country park No. 10, while 73.6%, 23%, and 3.4% of mildly ill patients are covered by the service scope of country parks Nos. 1, 8, and 11, respectively; In the robust optimization model, 100% of the critically ill patients in the designated treatment hospitals No. 4 were covered by the service scope of country park No. 10, with 63.2% and 36.8% of mildly ill patients being covered by the service scope of country parks Nos. 1 and 8, respectively. In addition, the coverage of patients varies when the same emergency medical facilities are allocated. For example, when the disturbance ratio is 5% and Γ = 7, in both the nominal model and the robust optimization model, all critically ill patients in the designated treatment hospital of No. 4 are covered by the service scope of country park No. 10, while mildly ill patients are covered by the service scope of country parks Nos. 1, 8, and 11. However, in the nominal model, 73.6%, 23%, and 3.4% of mildly ill patients are allocated to emergency medical facilities in country parks Nos. 1, 8, and 11 respectively, whereas in the robust optimization model, 61.4%, 33.6%, and 5.1% of mildly ill patients were assigned to emergency medical facilities in country parks Nos. 1, 8, and 11 respectively.

**Figure 7:**
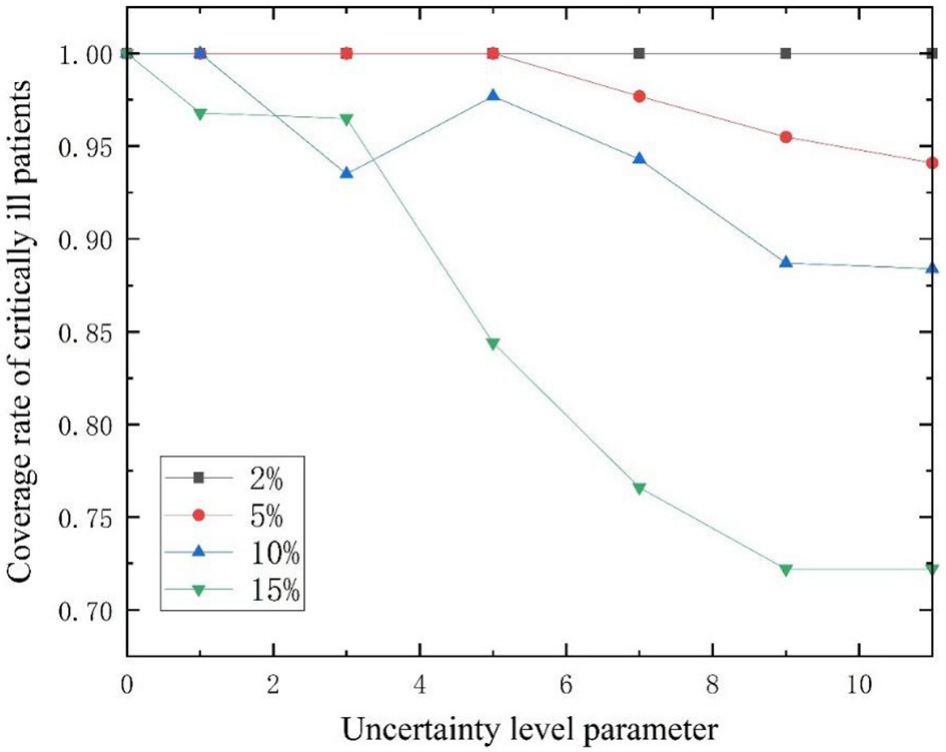
Changes in admission ratio of critically ill patients with different disturbance ratios as a function of Γ.

**Figure 8:**
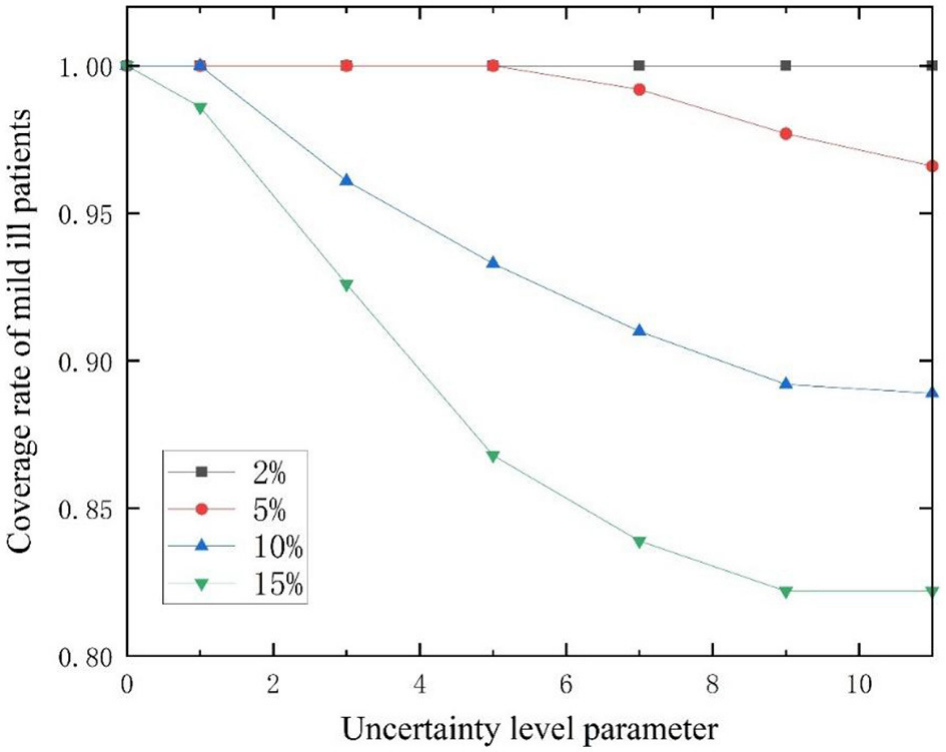
Changes in admission ratio of mildly ill patients with different disturbance ratios as a function of Γ.

In addition, the higher the disturbance ratio, the stronger the uncertainty. The disturbance ratios were modified to 2%, 5%, 10%, and 15% respectively (54). The variation of patient coverage ratio with uncertainty level parameter Γ* u*nder different disturbance ratios is shown in Figures 7, 8. The coverage ratio of critically ill patients decreases with the increase of uncertain level parameter Γ, and the er the disturbance ratio, the greater the degree of reduction. Among them, when the disturbance ratio is 10%, the coverage ratio of critically ill patients decreases first, then increases, and then decreases as the uncertainty level parameter Γ changes. This is because in the case of limited capacity, the model ensures that the total weighted service level of emergency medical facilities for all patients is the maximum. When the location plan changes, the coverage ratio of critically ill patients also changes. The coverage ratio of mildly ill patients decreases with the increase of uncertainty level parameter Γ, and the smaller the disturbance ratio, the smaller the reduction amplitude.

Under the same level of uncertainty, analyze the impact of different disturbance ratios on the location of emergency medical facilities and patient coverage (Tables 5–8). From Tables 5, 6, it can be seen that when the disturbance ratio is 2% and 5%, the site selection options are (10-1, 13-1, 1-2, 8-2, 11-2; indicating the establishment of rescue type emergency medical facilities in Country Parks 10 and 13, and monitoring type emergency medical facilities in Country Parks 1, 2, and 8), but the patient coverage varies.

**Table 5:** Coverage of emergency medical facility services at a disturbance ratio of 2% (Γ = 5).

**Designated treatment hospitals**	**Site selection plan and patient coverage**				
	**Critically ill patients**		**Mildly ill patients**		
	**10-1**	**13-1**	**1-2**	**8-2**	**11-2**
1	100%	0%	100%	0%	0%
2	100%	0%	0%	0%	100%
3	100%	0%	0%	0%	100%
4	100%	100%	69%	31%	0%
5	100%	0%	0%	0%	100%
6	0%	100%	0%	100%	0%
7	100%	100%	0%	100%	0%
8	0%	100%	0%	100%	100%
9	0%	100%	0%	100%	0%
10	0%	100%	0%	3.4%	96.6%
11	0%	100%	0%	100%	0%

**Table 6:** Coverage of emergency medical facility services at a disturbance ratio of 5% (Γ = 5).

**Designated treatment hospitals**	**Site selection plan and patient coverage**				
	**Critically ill patients**		**Mildly ill patients**		
	**10-1**	**13-1**	**1-2**	**8-2**	**11-2**
1	100%	0%	100%	0%	0%
2	100%	0%	0%	0%	100%
3	100%	0%	0%	0%	100%
4	100%	0%	63.2%	36.8%	0%
5	100%	0%	0%	0%	100%
6	0%	100%	0%	100%	0%
7	4%	96%	0%	100%	0%
8	0%	100%	0%	0%	100%
9	0%	100%	0%	0%	100%
10	0%	100%	0%	24.5%	75.5%
11	0%	100%	0%	100%	0%

**Table 7:** Coverage of emergency medical facility services at a disturbance ratio of 10% (Γ = 5).

**Designated treatment hospitals**	**Site selection plan and patient coverage**				
	**Critically ill patients**		**Mildly ill patients**		
	**8-1**	**11-1**	**1-2**	**7-2**	**10-2**
1	0%	79.2%	44.5%	0%	55.5%
2	0%	100%	0%	0%	0%
3	0%	100%	0%	0%	100%
4	55%	45%	54%	7%	39%
5	0%	100%	0%	0%	100%
6	100%	0%	100%	0%	0%
7	100%	0%	0%	100%	0%
8	0%	100%	0%	100%	0%
9	100%	0%	0%	100%	0%
10	66%	34%	0%	100%	0%
11	100%	0%	0%	100%	0%

**Table 8:** Coverage of emergency medical facility services at a disturbance ratio of 15% (Γ = 5).

**Designated treatment hospitals**	**Site selection plan and patient coverage**				
	**Critically ill patients**		**Mildly ill patients**		
	**8-1**	**11-1**	**1-2**	**7-2**	**10-2**
1	0%	19%	34%	0%	34%
2	0%	0%	0%	0%	0%
3	0%	100%	0%	0%	100%
4	50%	50%	49%	2%	49%
5	0%	100%	0%	38%	62%
6	100%	0%	75%	0%	0%
7	100%	0%	39%	61%	0%
8	0%	100%	0%	100%	0%
9	100%	0%	0%	100%	0%
10	40%	60%	0%	100%	0%
11	100%	0%	0%	100%	0%

**Figure 9:**
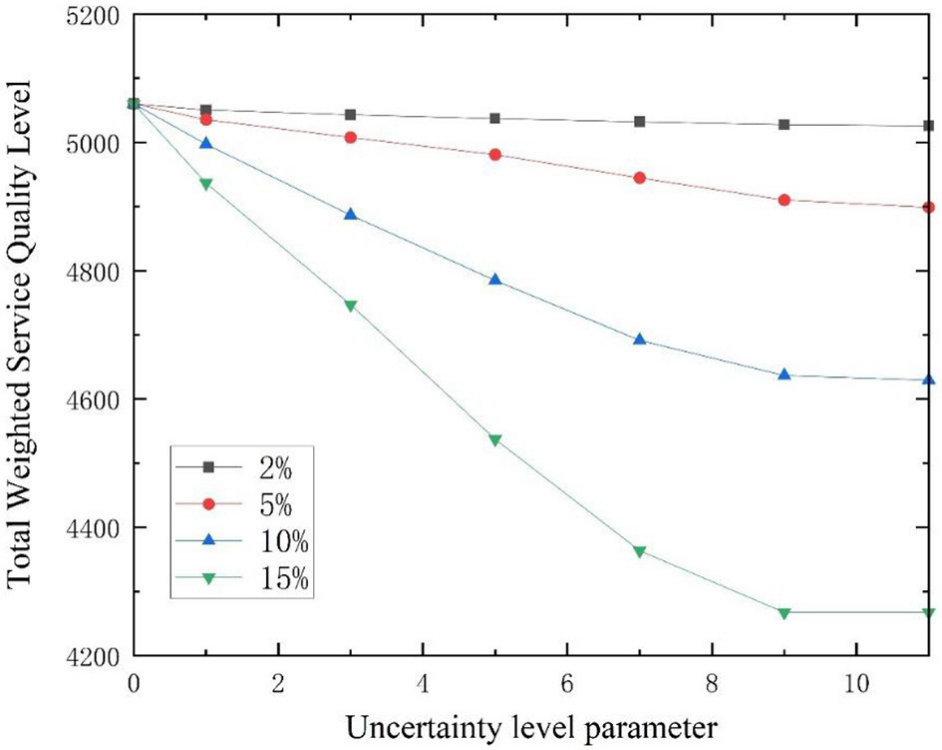
Changes in total weighted service quality level with uncertainty level.

When the uncertainty level Γ = 0, the robust optimization model (RM) under the polyhedral uncertainty set is equivalent to the nominal model (DM). At this point, the maximum weighted service quality level is 5060.129. Figure 9 shows the curve of the total weighted service quality level changing with the uncertainty level parameter Γ under different disturbance ratios. When 0 < Γ < 11, the total weighted service quality level decreases with the increase of uncertainty level Γ, and the larger the disturbance ratio, the greater the degree of reduction; when Γ≥11, the problem is equivalent to an absolute robust model, where the total weighted service quality level tends to remain unchanged, with a minimum value of 4267.355, and decreases by 0.7%, 3.2%, 8.5%, and 15.7% compared to *Z*^*D*^, respectively. The uncertain level parameter Γ_*k*_ = Γ is taken as 0, 1, 3, 5, 7, 9 and 11, respectively. The site selection schemes under different disturbance ratios and combinations of uncertain level parameters are analyzed, and the results are shown in Table 8. As the uncertain level parameter and disturbance ratio increase, the optimized site selection scheme will also change.

From Table 9, it can be seen that although the total weighted service quality levels vary under different combinations of uncertainty level parameters and disturbance ratios, there are only three site selection options (10–1, 13–1, 1–2, 8–2, 11–2), reflecting the model's good robustness and insensitivity to parameter disturbances. When the disturbance ratio is 10% and 9 ≤ Γ ≤ 11, the disturbance ratio is 10% and 7 ≤ Γ ≤ 11, the site selection scheme is (8–1, 11–1, 1–2, 6–2, 10–2), which can be chosen by conservative decision-makers; When the disturbance ratio is 15% and 3 ≤ Γ ≤ 5, the disturbance ratio is 10% and 5 ≤ Γ ≤ 7, the site selection scheme is (8–1, 11–1, 1–2, 7–2, 10–2), neutral decision-makers can choose this scheme; and when the disturbance ratio is 2%, 5% and 1 ≤ Γ ≤ 11, the disturbance ratio is 10% and 1 ≤ Γ ≤ 3, the site selection scheme is (10–1, 13–1, 1–2, 8–2, 11–2), which can be chosen by adventurous decision-makers.

**Table 9:** Site selection schemes under different disturbance ratios and uncertainty levels.

**Γ**	**Disturbance ratio**	**Total weighted service quality level**	**Site selection plan**
0	——	5060.129	10-1; 13-1; 1-2; 8-2; 11-2
1	2%	5050.47	10-1; 13-1; 1-2; 8-2; 11-2
	5%	5035.577	10-1; 13-1; 1-2; 8-2; 11-2
	10%	4997.366	10-1; 13-1; 1-2; 8-2; 11-2
	15%	4936.934	10-1; 13-1; 1-2; 8-2; 11-2
3	2%	5043.227	10-1; 13-1; 1-2; 8-2; 11-2
	5%	5007.954	10-1; 13-1; 1-2; 8-2; 11-2
	10%	4886.704	10-1; 13-1; 1-2; 8-2; 11-2
	15%	4747.216	8-1; 11-1; 1-2; 7-2; 10-2
5	2%	5037.173	10-1; 13-1; 1-2; 8-2; 11-2
	5%	4980.991	10-1; 13-1; 1-2; 8-2; 11-2
	10%	4784.79	8-1; 11-1; 1-2; 7-2; 10-2
	15%	4537.259	8-1; 11-1; 1-2; 7-2; 10-2
7	2%	5032.109	10-1; 13-1; 1-2; 8-2; 11-2
	5%	4944.796	10-1; 13-1; 1-2; 8-2; 11-2
	10%	4691.523	8-1; 11-1; 1-2; 7-2; 10-2
	15%	4363.688	8-1; 11-1; 1-2; 6-2; 10-2
9	2%	5027.714	10-1; 13-1; 1-2; 8-2; 11-2
	5%	4910.323	10-1; 13-1; 1-2; 8-2; 11-2
	10%	4636.612	8-1; 11-1; 1-2; 6-2; 10-2
	15%	4267.355	8-1; 11-1; 1-2; 6-2; 10-2
11	2%	5026.014	10-1; 13-1; 1-2; 8-2; 11-2
	5%	4898.935	10-1; 13-1; 1-2; 8-2; 11-2
	10%	4629.552	8-1; 11-1; 1-2; 6-2; 10-2
	15%	4267.355	8-1; 11-1; 1-2; 6-2; 10-2

## 4 Discussion

Through the analysis of the above results, we can obtain:

(1) Regarding the impact of uncertain factors on site selection, this article mainly considers the uncertainty of the number of different types of patients, referring to the Bertsimas and Sim robust optimization method. Specifically, the disturbance ratio and uncertainty level parameters have an impact on site selection decisions. The site selection decision plans obtained by combining different parameters will also be different (55).
(2) In the model, the uncertainty level parameter Γ and disturbance ratio are important parameters for adjusting the model. The size of Γ can reflect the decision-maker's decision-making preference, and the larger the value, the higher the decision-maker's tolerance for risk. Conversely, the lower the value. The disturbance ratio is an indicator used to measure the robustness of the model, thereby affecting the prediction accuracy and reliability of the model (56). Therefore, decision-makers need to choose an appropriate combination of uncertainty level parameter F and disturbance ratio based on their own risk preference to achieve the optimal decision result.
(3) Although the total weighted service quality of this model is influenced by the combination of uncertain level parameters and disturbance ratio, there are only three selection options, indicating that the model is less sensitive to changes in uncertain parameters. Even if the total weighted service quality corresponding to the exact selection option varies under different parameter combinations, it does not cause significant fluctuations in the entire decision plan, this indicates that the robustness of the model is relatively strong.

Previously, two-stage location selection method (57), Gray Wolf optimization algorithm (58), hierarchical and progressive location selection (59), P-center model (60) and overlay model (61) and other methods to analyze the improvement methods and rationality of facility allocation for emergency medical facility location. These methods are usually deterministic methods for optimization under known conditions, ignoring the uncertainties that may exist in the actual decision-making process (62). However, in the overall layout of emergency medical facilities, many uncertain factors need to be considered, such as the accessibility of emergency medical facilities, the support of surrounding infrastructure, and potential risks around (63). The decision scheme of the robust optimization model has greater optimality than that of the deterministic model (37, 64). In view of the uncertainty of public health emergencies, we choose to use the robust optimization theory to solve the location problem of emergency medical facilities, and incorporate country parks into the alternate sites for the construction of emergency medical facilities. The analysis results also support our idea. However, it should be noted that these country parks studied in this paper are all built for the purpose of leisure and recreation for urban residents, and their role in combating public health emergencies is rarely considered in the planning and design. Therefore, their site selection may not be the optimal location for building emergency medical facilities in the entire urban fringe.

Should we re-examine the types and functions of urban green Spaces? For example, in the suburbs of the city, we can select a suitable area for construction according to the site selection requirements of emergency medical facilities, and then carry out the planning, design and construction of park green space for combating health emergencies in this area. In “normal times,” it can provide city residents with the function of relaxing mood and releasing pressure; In “war time,” the park's infrastructure can be used to quickly set up emergency medical facilities. It can not only relieve the pressure on the medical system when the epidemic occurs, but also solve the problem of urban land use shortage. In this new planning and design, we can fully take into account the infrastructure required by the park, such as power, water supply, communication, etc., as well as the special facilities required by emergency medical facilities, such as isolation areas, temporary wards, etc. In this way, in the event of a public health emergency, the park can be quickly transformed into an emergency medical facility to provide emergency treatment for infected patients. In addition, for different emergency medical facility needs, we can also classify suburban parks according to size and function. For example, some larger country parks could accommodate more facilities and personnel to respond to a major outbreak; Some smaller country parks can be used as landing or dispersal sites for initial response and triage. On this basis, we also consider the uncertainty of the number of infected patients, and maximize the level of admission and treatment of emergency medical facilities, so as to provide a strong and sustainable public health response mechanism for cities to better cope with similar emergencies.

## 5 Conclusion

The COVID-19 has raised our concern and attention to urban public health safety. Urban public green space is not only a place for leisure and entertainment, but also an important resource to deal with public health emergencies. This article taking the function of urban public green space for disaster prevention and avoidance as the starting point, this paper discusses the problem of using country parks as the site selection of emergency medical facilities to deal with public health emergencies and the problem of preparing for and treating patients after the epidemic. Considering the uncertainty of the number of patients with different types of COVID-19, a polyhedral uncertainty set was introduced to characterize the uncertainty, and a maximum service quality level model was established based on the arrangement of two types of emergency medical facilities. Finally, on the basis of 13 selected country parks in Wuhan, the location of emergency medical facilities and patient transport scheme were determined, and the feasibility and effectiveness of the model were verified. The results show that compared with the traditional location selection method, this model can effectively promote the location selection scheme and patient transport route determination, and improve the utilization rate of medical resources. In addition, by optimizing the layout of emergency medical facilities, it can not only shorten the transport time of patients and reduce the risk of treatment delays, but also provide medical resource coverage for low-income groups and marginalized communities, and improve the equity and accessibility of emergency medical facilities.

However, this paper only considers the uncertainty of the number of confirmed patients, and in the actual situation, there are uncertainties in the driving speed and road conditions of the vehicle transporting patients, which need to be comprehensively analyzed and optimized. Future research can for all kinds of uncertainty factors in a more comprehensive analysis and optimization, in order to improve the efficiency and quality of public health emergency response. At the same time, more flexible and efficient emergency medical facility layout schemes can also be simulated by real scenarios or combined with case studies, such as setting mobile medical facilities in urban green Spaces, so as to adapt to the needs of various specific situations and provide better services and guarantees for urban emergency management.

## Data Availability

The original contributions presented in the study are included in the article/supplementary material, further inquiries can be directed to the corresponding author.
